# Time to Next Treatment Following Sub-Ablative Progression Directed Radiation Therapy for Oligoprogressive Non-Small-Cell Lung Cancer

**DOI:** 10.3390/curroncol31110505

**Published:** 2024-11-02

**Authors:** Riccardo Ray Colciago, Chiara Chissotti, Federica Ferrario, Maria Belmonte, Giorgio Purrello, Valeria Faccenda, Denis Panizza, Stefania Canova, Gaia Passarella, Diego Luigi Cortinovis, Stefano Arcangeli

**Affiliations:** 1 Medicine and Surgery Department, University of Milan Bicocca, 20126 Milano, Italy; riccardo.colciago@unimib.it (R.R.C.); chiarachissotti@gmail.com (C.C.); m.belmonte2@campus.unimib.it (M.B.); g.purrello@campus.unimib.it (G.P.); gaia.passarella@irccs-sangerardo.it (G.P.); diegoluigi.cortinovis@irccs-sangerardo.it (D.L.C.); stefano.arcangeli@unimib.it (S.A.); 2 Radiation Oncology Unit, Fondazione IRCCS San Gerardo dei Tintori, 20900 Monza, Italy; valeria.faccenda@irccs-sangerardo.it (V.F.); denis.panizza@irccs-sangerardo.it (D.P.); 3 Medical Physics Unit, Fondazione IRCCS San Gerardo dei Tintori, 20900 Monza, Italy; 4 Medical Oncology Unit, Fondazione IRCCS San Gerardo dei Tintori, 20900 Monza, Italy; stefania.canova@irccs-sangerardo.it

**Keywords:** progression directed radiation therapy, oligoprogression, non-small-cell lung cancer

## Abstract

We aimed to evaluate whether progression-directed radiation therapy (PDRT) can prolong the initiation of a subsequent systemic therapy regimen in a cohort of patients with oligoprogressive NSCLC. A retrospective analysis was conducted on NSCLC patients who underwent PDRT for extracranial oligoprogressive NSCLC, defined as limited (up to five) progressing lesions following initial complete, partial, or stable response to systemic therapy according to REC1ST 1.1 and/or PERCIST 1.0 criteria. Cox proportional hazard regressions were performed to identify factors influencing time to next treatment (TTNT), which was considered the primary endpoint. Forty patients were analyzed. First, second, and ≥3 lines of systemic therapy were administered in 22 (58.2%), 14 (27.2%), and 4 (14.6%) cases, respectively. The median total dose was 36 Gy (range: 12–60) in five fractions (1–10), with a median biological effective dose for tumor control (BED10) of 52 Gy (26.4–151.2). After a median follow-up of 11 months (2–50), PDRT delayed further systemic therapy in 32 (80.0%) treatments. Median TTNT was not reached at 8 months (1–47) with a one-year Kaplan–Meier estimate of 81.4% (95% CI: 75.0% to 87.8%). No >grade 3 adverse event was observed. On multivariate analysis, patients with ≥3 lines of systemic therapy and/or with larger CTV volumes did not benefit from PDRT. Despite the use of sub-ablative doses, our findings show that PDRT represents an effective, safe, and viable option for oligoprogressive NSCLC. Patients irradiated early during their systemic treatment course, with a low volume of disease and nonmetastatic oligoprogression, could derive substantial benefits from PDRT.

## 1. Introduction

Since the introduction of Hellman and Weichselbaum’s concept of oligometastatic disease [1], the conventional belief that metastatic disease equates to a poor prognosis has been challenged. Alongside advancements in systemic treatments, local ablative therapies such as surgery, radiation, and radiofrequency ablation have shown promising results in improving oncological outcomes [2,3,4]. Notably, evidence from a number of prospective randomized trials indicates a positive impact on progression-free survival (PFS) and overall survival (OS) across different tumor types [5,6,7,8,9,10]. Among other modalities, radiation therapy (RT) allows safe and noninvasive delivery of high doses of ionizing radiation in a few sessions, resulting in simultaneous treatment of multiple metastatic sites with minimal interruption of systemic therapy. While RT has shown promise in treating oligometastatic NSCLC, its effectiveness in the progressive or widespread metastatic setting remains unclear [11]. In the absence of biomarkers to identify the pattern of progressive disease, we currently rely on imaging or tissue biopsies to inform the therapeutic strategy. Progression after an initial response to systemic therapy is currently established as oligoprogression [12], a term first used in literature in 2012 to describe a clinical scenario where only a few metastases progress in a context of otherwise controlled metastatic disease [13]. The definition of oligoprogression is not widely accepted, with most studies considering three to five lesions as a cutoff for the definition [4,14]. Under these circumstances, the goal of RT is to prolong disease control and defer systemic treatment until the time of an overt progression. However, evidence remains limited, with most of the literature based on retrospective analyses. Understanding the correlation between established endpoints like progression-free survival (PFS), overall survival (OS), and time to next treatment (TTNT) may have relevant implications in the management of metastatic NSCLC. In this study, we aimed at evaluating the impact of a progression-directed RT (PDRT) on oncological outcomes in a cohort of patients with extracranial oligoprogressive NSCLC. Additionally, we sought to identify specific features associated with a higher likelihood of inducing a clinical–radiological response.

## 2. Materials and Methods

### 2.1. Study Design

We conducted a retrospective analysis at a single institution focused on patients diagnosed with NSCLC who received PDRT for extracranial oligoprogressive disease from May 2020 to December 2023. Oligoprogressive disease was defined as limited (up to 5) progressing lesions following initial complete, partial or stable response according to REC1ST 1.1 and/or PERCIST criteria [15,16]

### 2.2. Patient Selection

All patients included in the study had a prior diagnosis of NSCLC and had received standard of care (systemic therapy +/− RT to the primary site). During follow-up, patients underwent computed tomography (CT) scan and/or fluorodeoxyglucose positron emission tomography (FDG-PET) every three months. Each oligoprogressive case underwent a comprehensive evaluation by a multidisciplinary team, including radiation oncologists, medical oncologists, thoracic surgeons, radiologists, nuclear medicine physicians, and pathologists, to determine the optimal course of treatment.

Informed consent was obtained from all individual participants involved in the study.

### 2.3. Intervention

In eligible patients, targeted PDRT was administered to sites of oligoprogression with the aim of achieving durable local control while preserving the tolerance of surrounding organs at risk. The clinical target volume (CTV) was delineated following a review of the CT scans or FDG-PET images based on the multidisciplinary board’s discussion. An internal target volume (ITV) was obtained from a 4DCT scan when targets were located within organs affected by respiratory motion. The planning target volume (PTV) encompassed the CTV or ITV with a 3D margin determined and customized according to the treatment site. The dose prescription was planned to achieve the highest biologically effective dose using an α/β ratio of 10 (BED10), with an optimal goal of achieving 100 Gy. However, the dose was adjusted to preserve organ function and meet the dose-volume constraints. Treatment plans were optimized to ensure that the 95% isodose covered at least 95% of the PTV. If dose-volume constraints for a nearby organ at risk could not be respected, PTV coverage was compromised to prioritize organ preservation. In such cases, the treatment plans were optimized to ensure that the 95% isodose covered at least 95% of the CTV. PDRT was delivered using volumetric modulated arc therapy consisting of 6-FFF MV and 10-FFF MV coplanar arcs on a Linac platform. Accurate patient setup was ensured through kilovoltage cone-beam CT before each session to verify anatomical reproducibility.

### 2.4. Endpoints

The primary endpoint of the study was TTNT, defined as the time from the end of PDRT to the date of initiation of the first subsequent systemic therapy regimen, of the last follow-up or of the death [17]. This endpoint was considered met when a patient did not start a new systemic treatment—which would have been otherwise indicated—until the date of the last follow-up or to death. Secondary endpoints included local control (LC), PFS, and OS, defined as the time between the end of PDRT and local progression, local or distant progression, and death or of the last follow-up, respectively. The incidence of treatment-related adverse events was also measured.

### 2.5. Data Collection

Data on patient demographics, tumor histology, treatment modalities, response to systemic therapy, and outcomes were extracted from electronic medical records. Information regarding PDRT dose, fractionation, and target volumes was also collected. The toxicity profile was documented according to the common terminology criteria for adverse events (CTCAE) version 5.

### 2.6. Statistical Analysis

Univariate and multivariate Cox proportional hazard regressions were utilized to investigate the factors that correlate with the outcomes. The analysis encompassed patient demographics, tumor characteristics, treatment parameters, and systemic therapy details, assessed through Kaplan–Meier curves. Hazard ratios (HR) and 95% confidence intervals (CI) were computed to evaluate the association between these factors and the study endpoints. Factors with a *p*-value ≤ 0.10 in the univariate analyses were included in the multivariate Cox regression model. MedCalc^®^ v22.021 (MedCalc Software Ltd., Ostend, Belgium) was used for all statistical analyses. A significance level of *p* ≤ 0.05 was used to determine statistical significance.

## 3. Results

### 3.1. Population Description

Forty patients were analyzed. Patient and tumor characteristics are summarized in Table 1; treatment characteristics are displayed in Table 2. The median age of the whole cohort was 69 years (range: 43–87). Gender distribution was fairly balanced, with 45% males and 55% females. Smoking habits within the cohort were diverse, with 15.0% classified as nonsmokers, 22.5% as active smokers, and the majority, 62.5%, as former smokers, demonstrating a median pack/year of 25 (range: 0–100). Histologically, 92.5% of cases presented with adenocarcinoma, while the remaining 7.5% were diagnosed with squamous cell carcinoma. Molecular analysis revealed notable mutational statuses, with 55.0% of patients exhibiting any PDL1 IHC positivity, 45.0% demonstrating at least one oncogenic driver mutation, 30.0% displaying both oncogenic mutations and PDL1 positivity and 12.5% showing neither oncogenic driver mutations nor PDL1 expression. At the time of PDRT, every patient had a stage IV disease. The systemic therapy distribution preceding PDRT was as follows: 22 (55.0%) PDRT were added to first-line therapy, 14 (35.0%) were given during second-line therapy, and 4 (10.0%) were administered during third-line or subsequent therapies. Regarding systemic therapy types prior to PDRT, 12 (30.0%) patients underwent chemotherapy, 19 (47.5%) received immunotherapy (including two who received chemo-immunotherapy), and nine (22.5%) were treated with targeted therapy. At the last imaging evaluation, seven (17.5%), 23 (57.5%), and 10 (25%) achieved complete response, partial response, and stable disease, respectively, according to RECIST and/or PERCIST criteria to all the other sites of the disease.

### 3.2. Treatment Description

PDRT was administered to different anatomical sites: 17 (42.5%) treatments were focused on the primary tumor, nine (22.5%) on regional lymph nodes, and 14 (35.0%) on distant metastases. The median dose of PDRT was 36 Gy (range: 12–60), delivered over a median of five fractions (range: 1–10), with a median biologically effective dose for tumor control (BED10) of 52 Gy (range: 26.4–151.2). Seven patients (17.5%) had previously undergone chemoradiotherapy for their primary tumor, with a median dose of 60 Gy in 30 fractions. Of these, one received PDRT on the primary tumor after 48 months, and two received PDRT on regional nodal disease 12 and 43 months after the initial radiation treatment, respectively.

As reported in Table 3, schedules varied depending on the treatment site. RT to the primary tumor covered a range of doses from 30 to 60 Gy in 3 to 10 fractions. The median BED10 was 65.2 Gy, ranging from 39 to 151.2 Gy. Nodal irradiation schedules varied from 20 Gy in five fractions to 30 Gy in five fractions and a median BED10 of 39 Gy (28–48). RT at the level of metastatic sites was more heterogeneous, with a range of 12–25 Gy in 1–5 fractions (BED10 26.4–43.2 Gy), 20–45 Gy in 5–8 fractions (BED10 28–78.7 Gy), and 24–60 Gy in 3–8 fractions (BED10 43.2–105 Gy) on bone, renal/adrenal, and contralateral lung metastases, respectively.

The median volume of the CTV and PTV was 22 cc (range: 0.5–192.5) and 50.5 cc (range: 4.7–411.9), respectively. The median coverage of the CTV was 100% for the volume receiving 95% of the prescribed dose (V95%), with a median value of 99.7% for the volume receiving 99% of the dose (V99%). Similarly, the PTV had a median coverage of 96.7% for V95%, with a median value of 80.2% for V99%.

### 3.3. Treatment Outcomes

The median follow-up duration for the cohort was 11 months (range: 2–50). PDRT delayed the initiation of a new line of systemic therapy in 80.0% of cases, with a median TTNT not reached at 7 months (range: 1–47). The one-year Kaplan–Meier estimate for TTNT was 81.4% (95% CI: 75.0% to 87.8%). In the Cox univariate analysis, receiving systemic target therapy (HR = 7.3, 95% CI: 1.20 to 45.20; *p* = 0.03) and nonsmoking (HR = 6.62, 95% CI: 1.30 to 33.6; *p* = 0.02) were identified as a risk factor affecting treatment outcomes. Gender, age, and CTV coverage (HR 0.15, *p* = 0.08; HR 0.93, *p* = 0.10; HR 0.82, *p* = 0.10) showed a trend to correlation indicating a lower chance of change in systemic therapy for older male patients with a higher V95% of the CTV. However, in the Cox multivariate analysis, no variable showed a significant association with TTNT.

Table 4 presents the results of the univariate and multivariate analysis for TTNT.

Six treated sites underwent local progression, accounting for 15.0% of cases. Among them, three were at the regional nodal level, two were at the primary tumor, and one was at distant sites. The median LC was not reached at 8 months, ranging from 1 to 44 months. The one-year Kaplan–Meier estimate for LC was 79.6%, with a 95% CI of 72.2% to 88.0%. Target coverage was associated with better local control at univariate analysis: HR = 0.62 (95% CI: 0.41 to 0.94; *p* = 0.02). Age and nonsmoking habits showed just a trend of correlation with better disease control without reaching the significance cut-off (*p* = 0.06 and 0.07, respectively). However, no variables demonstrated a significant association with LC in the multivariate analysis. Table 5 summarizes variables that were below the cut-off of *p* ≤ 0.10 in the univariate analysis for LC, DFS, and OS.

Disease progression occurred in 19 cases (47.5%). The median DFS was not reached at 5 months, ranging from 0 to 21 months, with a one-year Kaplan–Meier of 47.5% (95% CI: 38.0% to 56.0%). In the Cox univariate analysis, significant associations were found between target coverage (HR = 0.79, 95% CI: 0.64 to 0.98; *p* = 0.03) and pack/year smoking history (HR = 0.97 for one pack/year increase, 95% CI: 0.95 to 0.99; *p* = 0.02) as protective factors. Complete response as per RECIST and/or PERCIST criteria in all the other sites of disease showed a trend to significance in favor of better disease control when compared to partial or stable response (HR = 0.15, 95% CI: 0.02 to 1.2; *p* = 0.07). In the Cox multivariate analysis, they all confirmed their correlation as protective factors for DFS (HR = 0.74 *p* = 0.02, HR = 0.96 *p* = 0.02, and HR = 0.011 *p* = 0.04, respectively).

Ten deaths occurred, representing 25.0% of cases. The median OS was not reached at 10 months (range 1–48) months. The one-year OS actuarial rate was 81.5%, with a 95% CI of 74.6% to 88.4%. In the Cox univariate analysis, receiving systemic therapy lines ≥ 3 (HR = 4.88, 95% CI: 1.09 to 21.80; *p* = 0.03) and CTV volume (HR = 1.01 for a 1cc increase, 95% CI: 1.001 to 1.02; *p* = 0.06) was associated with worse OS. In the Cox multivariate analysis, both systemic therapy line ≥ 3 (HR = 7.85, 95% CI: 1.41 to 43.49; *p* = 0.02) and CTV volume (HR = 1.01 for a 1cc increase, 95% CI: 1.001 to 1.02; *p* = 0.03) remained significant factors associated with worse OS.

**Figure 1:**
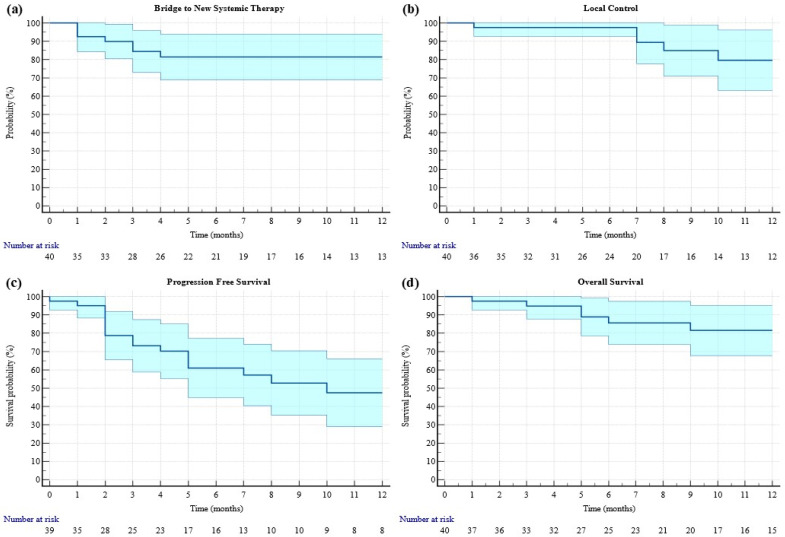
One-year Kaplan–Meier for time to next treatment, local control, progression-free survival and overall survival. (**a**) Time to next systemic therapy: median 7 (range 1–47) months; (**b**) local control: median 8 (range 1–44) months; (**c**) progression-free survival: median 5 (range 0–21) months; (**d**) overall survival: 10 (range 1–48) months.

Figure 1 summarizes the one-year Kaplan–Meier estimates for each endpoint.

Toxicity was observed in 10 cases, accounting for 25.0% of the total cohort, with seven cases (17.5%) classified as Grade (G) 1 and three cases (7.5%) as G2, while no ≥G2 toxicity was documented. All recorded toxicity events were identified as radiation pneumonitis. Among the three patients who underwent a second course of radiation, only one, who was irradiated at the level of regional lymph nodes, experienced grade 1 radiation pneumonitis.

## 4. Discussion

The evolving landscape of systemic oncological therapies, coupled with modern imaging, has led to the increasing recognition of oligoprogression. Evidence on the optimal treatment approach remains limited [18]. In our series of 40 patients, with a median follow-up of 11 months (range: 2–50), we found that PDRT achieved high rates of LC (85.0%) in the absence of major toxicities, and was associated with median TTNT, DFS, and OS not reached at 7 months (range: 1–47), 5 months (range: 0–21), and 10 months (range: 1–48), respectively. In contrast, very few side effects were observed in our cohort, with only three patients (7.5%) experiencing G2 toxicities and no severe toxicities. Franceschini et al. [19] reported that RT, when added to the SOC, in oligoprogressive NSCLC, could achieve DFS ranging from 5.5 to 10.9 months. However, Mavrikios et al. [11] conducted a literature review and found that the majority of studies are retrospective and focused on specific types of systemic therapy. Tsai et al. [20] reported the results of the first phase 2 trial in which patients with oligoprogressive breast cancer and NSCLC were randomized to receive either SOC or SBRT to the progression site plus SOC. The most common RT dose schedules were 27–30 Gy in three fractions and 30–50 Gy in five fractions, accounting for 71% of the treatments. In a subgroup analysis, patients with oligoprogressive NSCLC (28 in the SOC group and 31 in the RT group) experienced significantly longer DFS in the experimental arm [10.0 months vs. 2.2 months; Hazard Ratio (HR) 0.41, *p* = 0.0039]. However, 34 patients (62%) experienced ≥ G2 toxicities, including three cases of G4 toxicity. Schellenberg et al. [21] randomized 90 oligoprogressive patients (44% with lung cancer) to receive either SOC or SBRT plus SOC. Oligoprogression was defined as 1–5 actively progressing lesions during systemic therapy. SBRT was delivered in 1–5 fractions, with a total dose ranging from 16 to 55 Gy. The trial showed no significant differences between the experimental and standard arms in terms of DFS (8.4 vs. 4.3 months, *p* = 0.91) and OS (31.2 vs. 27.4 months, *p* = 0.22). SBRT did not improve TTNT (10.3 vs. 7.6 months, *p* = 0.71), but LC was significantly higher in the SBRT arm compared to the SOC arm (71% vs. 39%, *p* = 0.002). Major side effects (> G2) were experienced by two patients (3.3%) in the SBRT arm.

Our findings are difficult to compare with the aforementioned studies, primarily due to differences in dose prescriptions. In our cohort, the median PDRT dose was 36 Gy (range: 12–60), delivered over a median of five fractions (range: 1–10), with a median BED10 of 52 Gy (range: 26.4–151.2), which is lower than the SBRT doses used elsewhere. We acknowledge that the optimal PDRT dose and fractionation adopted in our study might be arguable. Prospective studies have used BED10 doses of 100 Gy with an ablative intent [22,23], and there is increasing evidence supporting the use of single-fraction schedules [24,25], depending on tumor size and location. However, in the context of oligoprogression, PDRT was thought to prolong response to systemic therapy by eradicating nonsensitive dedifferentiated clones [26]. Therefore, the goal of the management of oligoprogression with PDRT was to reduce the burden of treatment-resistant cells in order to be able to continue systemic therapy, postponing further treatments until the onset of overt metastatic progression. To achieve this objective, ablative radiation doses, which could increase toxicity and negatively impact the quality of life, may not always be necessary. In our cohort, we opted for organ-preserving doses of stereotactic radiation to treat sites of oligoprogression, demonstrating that tailored treatments can achieve optimal disease control with minimal toxicity. Supporting this approach, we reference the study by Mahmood et al. [27]. In their retrospective study, Mahmood et al. analyzed 120 patients, 59 of whom had lung cancer, who experienced progression while receiving immunotherapy. These patients were treated with a wide range of radiation therapies, from conventional schedules to stereotactic approaches. The study reported favorable rates of PFS (median 6.4 months) and OS (median 29.8 months), and the use of sub-ablative doses did not show a statistically significant difference in disease control compared to higher doses.

Another retrospective analysis was conducted by Ebadi et al. [28]. They treated a total of 168 oligoprogressive or oligorecurrent NSCLC patients, with the addition of SBRT to systemic treatment. Dose schedules are comparable to ours as they used a median of 30 Gy (15–60) in five fractions (1–5), resulting in a median BED10 of 48 Gy. With a median follow-up of 17 months, the median TTNT, PFS and OS were 6.6, 9, and 31 months, respectively. Grade 3 toxicity was seen in three (1.8%) patients.

While our TTNT results are comparable, PFS and OS were slightly inferior, possibly due to the shorter follow-up in our study. Nevertheless, to achieve better outcomes, patient selection appears to be a key factor. In our cohort, 45.0% of patients received more than one line of systemic therapy prior to PDRT, compared to those experiencing oligoprogression after only the first line of treatment. In fact, receiving three or more lines of systemic therapy was associated with poorer OS outcomes. In our cohort, we observed that patients who benefited most from PDRT were those treated early in their systemic therapy course and who had a low disease burden. Several studies have confirmed a correlation between the line of therapy and oncological outcomes [29]. Kroeze et al. [30] treated 108 NSCLC patients, of whom 56% were oligoprogressive, with systemic therapy + PDRT. After a median follow-up of 18.7 months, PFS was 8.7 months. Previous lines of systemic therapy and the site of disease were significant predictors of PFS, as confirmed in the multivariate analysis (HR 1.7; *p*  =  0.033). Similar results were reported by Mok et al. [31], who treated 55 oligoprogressive NSCLC patients with local ablative radiation therapy, with a BED10 ranging from 37.5 (to the brain) to 151.2 (to the lung). The median follow-up time was 13.3 months (range: 1.5–51.6 months). The median PFS2 was 6.9 months (95% CI: 3.1 to 10.7 months). In the multivariate analysis, worse PFS was associated with receiving more lines of prior systemic therapy (*p* = 0.001). These results were further demonstrated more recently in the phase 2 CURB study, where the breast cancer population with oligoprogressive disease had undergone more advanced lines of systemic therapy (median of 3rd to 4th line) compared to the NSCLC cohort (median of 2nd line), thus potentially reflecting that more advanced and refractory disease may impact the effectiveness of PDRT strategy [20].

Interestingly, a correlation was found between the volume of the CTV and OS: for every one cc increase of the CTV, OS decreased with an HR of 1.01 (*p* = 0.03). Similar results are reported in a large cohort of oligometastatic patients by Yamamoto et al. [32]. They analyzed 1378 patients with different oligometastatic tumors and found a correlation between the CTV diameter and OS (per 1-cm increase; HR, 1.266; 95% CI: 1.131 to 1.417; *p*  <  0.001). The LaIT-SABR study [33] analyzed 1033 lung oligometastases and showed that lesions with a diameter > 20 mm are related to worse DFS.

Unexpectedly, the number and location of PDRT sites did not show any correlation with worse DFS. Patients treated on multiple or metastatic lesions did not exhibit a trend toward increased disease progression compared to those treated on nodal or tumoral sites. Although few studies focused on oligoprogressive NSCLC, others revealed a close correlation between the site of the disease and the oncological outcomes [22,34]: Franzese et al. [34] analyzed 437 oligometastases of 270 colorectal cancer patients treated with SBRT. In their cohort, nonlung metastases were related to significantly worse OS (HR 1.67; *p* = 0.02). Franceschini et al. [22] showed similar results in their sample of 358 oligometastatic patients treated with SBRT, with nodal metastases associated with longer OS (HR 0.44; *p* = 0.005). Regarding the number of lesions, the series by Ebadi et al. [28] indicated that patients with three to five sites of metastatic progression had worse OS (HR = 2.6, 95% CI: 1.5 to 4.3, *p* < 0.001) and shorter TTNT (HR = 1.7, 95% CI: 1.1 to 2.5, *p* = 0.01) compared to those with zero to two sites.

Finally, the complete response of the tumor to prior systemic therapy may play a key role in patient prognosis and could be crucial for patient selection. We found that complete response (CR) is associated with better PFS (HR = 0.11, 95% CI: 0.01 to 0.97; *p* = 0.04 in the multivariate analysis). This variable was also linked to improved disease control in the meta-analysis by Rosner et al. [35], where CR was shown to enhance both OS and PFS with HRs of 0.50 (95% CI: 0.45 to 0.56) and 0.46 (95% CI: 0.37 to 0.57), respectively.

### Study Limitations

We acknowledge several limitations in this study. Its retrospective nature is inherently associated with biases. Moreover, the sample size is limited, and the median follow-up duration is relatively short at 11 months. Certainly, a larger number of patients and a longer follow-up could be useful for a more in-depth analysis. Nevertheless, oligoprogressive NSCLC has a rapid evolution, and outcomes at 1 year after PDRT are meaningful. In addition, the impact of such an approach on QoL has not been documented, which is increasingly relevant given the widespread use of less toxic systemic treatments such as molecularly targeted agents and immunotherapy. Furthermore, the enrollment of patients with different genomic characteristics (i.e., wild type vs. driver mutations) may have confounded the final results about local and distant disease control due to different biological disease behavior, which could explain the difference between TTNT and DFS in our cohort.

Despite these limitations, we believe that our findings provide interesting data on the impact of local therapy for systemic control in a disease whose pattern of metastatic spread is often limited.

## 5. Conclusions

Despite the use of sub-ablative doses, our findings demonstrate that PDRT is an effective, safe, and viable option for oligoprogressive NSCLC. Patients irradiated early during their systemic treatment course, with a low volume of disease could derive substantial benefits from PDRT. Mature data from randomized controlled trials are awaited to confirm whether the deferral of systemic treatment until the time of progression via PDRT concerning a consolidative treatment on the residual burden of “oligometastatic disease” during a systemic therapy may improve clinical outcomes in this NSCLC patient’s subgroups.

## Figures and Tables

**Table 1:** Patients and tumor.

Age (Years)	
**Median**	69 (Range: 43–87)
**Sex**	
Male	18 (45.0%)
Female	22 (55.0%)
**Smoking habit**	
Never smokers	6 (15.0%)
Active smokers	9 (22.5%)
Former smokers	25 (62.5%)
**Histology**	
Adenocarcinoma	37 (92.5%)
Squamous cell carcinoma	3 (7.5%)
**Oncogenic driver mutations**	
Mutated:	18 (45.0%)
KRAS	6 (15.0%)
EGFR	4 (10.0%)
ALK	3 (7.5%)
BRAF	1 (2.5%)
ROS1	1 (2.5%)
Other	3 (7.5%)
Absence	22 (45.0%)
**PDL1 status**	
Positive:	22 (55%)
*<50%*	*7 (17.5%)*
*≥50%*	*15 (37.5%)*
Negative:	18 (45%)

**Table 2:** Treatment characteristics.

Pre-RT systemic therapy regimens	
I	22 (55.0%)
II	14 (35.0%)
≥III	4 (10.0%)
**Type of pre-RT systemic therapy**	
Chemotherapy	10 (25.0%)
Immunotherapy	19 (47.5%)
Chemo-immunotherapy	2 (5.0%)
Targeted	9 (22.5%)
**Response to systemic therapy**	
Complete	7 (17.5%)
Partial	23 (57.5%)
Stable	10 (25.0%)
**Number of synchronous PDRT**	
1	35 (87.5%)
2	5 (12.5%)
**RT site**	
Primary	17 (42.5%)
Regional lymph nodes	9 (22.5%)
Metastatic	14 (35.0%)
Bone	5 (12.5%)
Contralateral lung	4 (10%)
Adrenal gland	4 (10%)
Kidney	1 (2.5%)
**CTV (cc)**	
Median	22 (range: 0.5–192.5)
**PTV (cc)**	
Median	50.5 (range: 4.7–411.9)
**RT dose (Gy)**	
Median	36 (range: 12–60)
**Number of fractions**	
Median	5 (range: 1–10)
**BED_10_**	
Median	52 (range: 26.4–151.2)

RT: radiation therapy; PDRT: metastases-directed radiation therapy; CTV: clinical target volume; PTV: planning target volume; BED_10_: biologically effective dose considering an α/β = 10.

**Table 3:** Dose prescription.

Site	Volume (cc) of CTV	Total Dose	Fractions	BED_10_
*Adrenal Gland*	157.0	20	5	*28.0*
*Adrenal Gland*	5.4	30	5	*48.0*
*Adrenal Gland*	10.8	40	8	*60.0*
*Adrenal Gland*	3.4	45	6	*78.8*
*Bone*	85.4	12	1	*26.4*
*Bone*	27.8	21	3	*35.7*
*Bone*	23.2	25	5	*37.5*
*Bone*	6.9	16	1	*41.6*
*Bone*	62.9	24	3	*43.2*
*Kidney*	25.1	30	5	*48.0*
*Lung*	142.8	30	10	*39.0*
*Lung*	36.5	30	10	*39.0*
*Lung*	10.4	24	3	*43.2*
*Lung*	134.4	30	5	*48.0*
*Lung*	2.7	36	6	*57.6*
*Lung*	38.2	45	10	*65.3*
*Lung*	20.8	45	9	*67.5*
*Lung*	30.5	50	10	*75*
*Lung*	192.5	50	10	*75*
*Lung*	6.3	45	6	*78.8*
*Lung*	61.8	45	6	*78.8*
*Lung*	38.5	45	6	*78.8*
*Lung*	31.1	45	6	*78.8*
*Lung*	25.8	45	5	*85.5*
*Lung*	24.1	48	6	*86.4*
*Lung*	2.7	50	5	*100.0*
*Lung*	16.9	60	8	*105.0*
*Lung*	9.8	60	8	*105.0*
*Lung*	0.5	45	3	*112.5*
*Lung*	71.8	45	3	*112.5*
*Lung*	5.7	54	3	*151.2*
*Regional Lymph Node*	3.3	20	5	*28.0*
*Regional Lymph Node*	12.7	25	5	*37.5*
*Regional Lymph Node*	3.0	25	5	*37.5*
*Regional Lymph Node*	172.6	30	10	*39.0*
*Regional Lymph Node*	4.7	24	3	*43.2*
*Regional Lymph Node*	0.7	24	3	*43.2*
*Regional Lymph Node*	1.7	30	5	*48.0*
*Regional Lymph Node*	71.1	30	5	*48.0*
*Regional Lymph Node*	3.7	40	10	*56.0*

**Table 4:** Univariate and multivariate Cox hazard regression for time to next therapy.

	*Variable*	HR	95% CI	*p*-Value
***Univariate***	***No smoking habit***	6.62	1.30–33.62	**0.02**
	***Target therapy***	7.39	1.20–45.21	**0.03**
	*Male gender*	0.15	0.01–1.28	0.08
	*Age for 1 year increase*	0.93	0.86–1.01	0.10
	*V_ctv95%_*	0.82	0.65–1.04	0.10
	Stable response to therapy	0.30	0.03–2.45	0.26
	No mutations	2.80	0.32–23.91	0.34
	BED_10_	0.98	0.95–1.01	0.41
	Multiple PDRT	1.75	0.34–8.98	0.49
	CTV volume	1.00	0.99–1.01	0.51
	Metastatic site of PDRT	1.73	0.28–10.39	0.54
	>2 lines of systemic therapy	1.63	0.18–14.69	0.66
**Multivariate**	*None*	-	-	*-*

HR: hazard ratio; CI: confidence interval; BED_10_: biologically effective dose considering an α/β = 10; V_ctv95%_: volume of the clinical target volume receiving the 95% of the prescribed dose.

**Table 5:** Univariate and multivariate Cox hazard regression for local control, progression-free survival and overall survival.

		Variable	HR	95% CI	*p*-Value
**Local control**					
	**Univariate**				
		**V_ctv95%_**	0.62	0.41–0.94	**0.02**
		Age	0.88	0.70–1.01	0.06
		No smoking habit	5.89	0.81–42.12	0.07
	**Multivariate**	None	-	-	*-*
**Progression-free survival**					
	**Univariate**				
		Pack/year	0.97	0.94–0.99	**0.02**
		V_ctv95%_	0.79	0.64–0.98	**0.03**
		Complete response to therapy	0.15	0.02–1.20	0.07
	**Multivariate**	Pack/year	0.95	0.92–0.98	**0.005**
		Vctv95%	0.73	0.55–0.96	**0.02**
		Complete response to therapy	0.11	0.01–0.97	**0.04**
**Overall survival**					
	**Univariate**				
		≥3 lines of systemic therapy	4.88	1.09–21.8	**0.03**
		CTV Volume	1.01	1.001–1.02	**0.04**
	**Multivariate**				
		≥3 line of systemic therapy	7.85	1.41–43.49	**0.02**
		CTV volume	1.01	1.001–1.02	**0.03**

HR: hazard ratio; CI: confidence interval; V_ctv95%_: volume of the clinical target volume receiving 95% of the prescribed dose; CTV: clinical target volume.

## Data Availability

The datasets used and/or analyzed during the current study are available from the corresponding author upon reasonable request.

## References

[1] Hellman S., Weichselbaum R.R. (1995). Oligometastases. J. Clin. Oncol..

[2] Fong Y., Fortner J., Sun R.L., Brennan M.F. (1999). Clinical score for predicting recurrence after hepatic resection for metastatic colorectal cancer: Analysis of 1001 consecutive cases. Ann. Surg..

[3] Pastorino U., Buyse M., Friedel G. (1997). International Registry of Lung Metastases. Long-term results of lung metastasectomy: Prognostic analyses based on 5206 cases. J. Thorac. Cardiovasc. Surg..

[4] Guckenberger M., Lievens Y., Bouma A.B. (2020). Characterisation and classification of oligometastatic disease: A European Society for Radiotherapy and Oncology and European Organisation for Research and Treatment of Cancer consensus recommendation. Lancet Oncol..

[5] Ruers T., Van Coevorden F., Punt C.J., European Organisation for Research and Treatment of Cancer (EORTC), Gastro-Intestinal Tract Cancer Group, Arbeitsgruppe Lebermetastasen und tumoren in der Chirurgischen Arbeitsgemeinschaft Onkologie (ALM-CAO), National Cancer Research Institute Colorectal Clinical Study Group (NCRI CCSG) (2017). Local Treatment of Unresectable Colorectal Liver Metastases: Results of a Randomized Phase II Trial. J. Natl. Cancer Inst..

[6] Ost P., Jereczek-Fossa B.A., As N.V., Zilli T., Muacevic A., Olivier K., Henderson D., Casamassima F., Orecchia R., Surgo A. (2016). Progression-free survival following stereotactic body radiotherapy for oligometastatic prostate cancer treatment-naive recurrence: A multi-institutional analysis. Eur. Urol..

[7] Phillips R., Shi W.Y., Deek M., Radwan N., Lim S.J., Antonarakis E.S., Rowe S.P., Ross A.E., Gorin M.A., Deville C. (2020). Outcomes of observation vs stereotactic ablative radiation for oligometastatic prostate cancer: The ORIOLE phase 2 randomized clinical trial. JAMA Oncol..

[8] Iyengar P., Wardak Z., Gerber D.E., Tumati V., Ahn C., Hughes R.S., Dowell J.E., Cheedella N., Nedzi L., Westover K.D. (2018). Consolidative radiotherapy for limited metastatic non-small-cell lung cancer: A phase 2 randomized clinical trial. JAMA Oncol..

[9] Palma D.A., Olson R., Harrow S., Gaede S., Louie A.V., Haasbeek C., Mulroy L., Lock M., Rodrigues G.B., Yaremko B.P. (2020). Stereotactic ablative radiotherapy for the comprehensive treatment of oligometastatic cancers: Long-term results of the SABR-COMET phase II randomized trial. J. Clin. Oncol..

[10] Gomez D.R., Tang C., Zhang J., Blumenschein G.R., Hernandez M., Lee J.J., Ye R., Palma D.A., Louie A.V., Camidge D.R. (2019). Local consolidative therapy vs. maintenance therapy or observation for patients with oligometastatic non-small-cell lung cancer: Long-term results of a multi-institutional, phase II, randomized study. J. Clin. Oncol..

[11] Mavrikios A., Remon J., Quevrin C., Mercier O. (2023). Local control strategies for management of NSCLC with oligoprogressive disease. Cancer Treat. Rev..

[12] Xu Y., Li H., Fan Y. (2021). Progression Patterns, Treatment, and Prognosis Beyond Resistance of Responders to Immunotherapy in Advanced Non-Small Cell Lung Cancer. Front. Oncol..

[13] Weickhardt A.J., Scheier B., Burke J.M. (2012). Local ablative therapy of oligoprogressive disease prolongs disease control by tyrosine kinase inhibitors in oncogene-addicted non-small-cell lung cancer. J. Thorac. Oncol..

[14] Patel P.H., Palma D., McDonald F. (2019). The Dandelion Dilemma Revisited for Oligoprogression: Treat the Whole Lawn or Weed Selectively?. Clin. Oncol..

[15] Eisenhauer E.A., Therasse P., Bogaerts J., Schwartz L.H., Sargent D., Ford R., Dancey J., Arbuck S., Gwyther S., Mooney M. (2009). New response evaluation criteria in solid tumours: Revised RECIST guideline (version 1.1). Eur. J. Cancer.

[16] Lodge M.A., Wahl R.L. (2016). Practical PERCIST: A Simplified Guide to PET Response Criteria in Solid Tumors 1.0. Radiology.

[17] Marshall J., Schwartzberg L.S., Bepler G., Spetzler D., El-Deiry W.S., Xiao N., Reddy S.K., Kim E.S., Poste G.H., Raghavan D. (2016). Novel panomic validation of time to next treatment (TNT) as an effective surrogate outcome measure in 4729 patients. J. Clin. Oncol..

[18] Hanna G.G., McDonald F. (2024). SBRT for oligoprogressive disease: Using the evidence to maximise the benefits. Lancet.

[19] Franceschini D., De Rose F., Cozzi S. (2020). The use of radiation therapy for oligoprogressive/oligopersistent oncogene-driven non-small cell lung cancer: State of the art. Crit. Rev. Oncol. Hematol..

[20] Tsai C.J., Yang J.T., Shaverdian N., CURB Study Group (2024). Standard-of-care systemic therapy with or without stereotactic body radiotherapy in patients with oligoprogressive breast cancer or non-small-cell lung cancer (Consolidative Use of Radiotherapy to Block [CURB] oligoprogression): An open-label, randomised, controlled, phase 2 study. Lancet.

[21] Schellenberg D., Gabos Z., Duimering A. (2024). Stereotactic Ablative Radiotherapy for Oligoprogressive Cancers: Results of the Randomized Phase II STOP Trial. Int. J. Radiat. Oncol. Biol. Phys..

[22] Franceschini D., De Rose F., Franzese C. (2019). Predictive Factors for Response and Survival in a Cohort of Oligometastatic Patients Treated with Stereotactic Body Radiation Therapy. Int. J. Radiat. Oncol. Biol. Phys..

[23] Ratnakumaran R., McDonald F. (2022). The Management of Oligometastases in Non-small Cell Lung Cancer—is Stereotactic Ablative Radiotherapy Now Standard of Care?. Clin. Oncol..

[24] Cuccia F., Mazzola R., Figlia V., Giaj-Levra N., Nicosia L., Ricchetti F., Rigo M., Attinà G., Vitale C., Pastorello E. (2022). Stereotactic body radiotherapy for pulmonary oligometastases: A monoinstitutional analysis of clinical outcomes and potential prognostic factors. Strahlenther. Onkol..

[25] Greenwood H., Hassan J., Fife K., Ajithkumar T.V., Thippu Jayaprakash K. (2023). Single-Fraction Stereotactic Ablative Body Radiotherapy for Primary and Extracranial Oligometastatic Cancers. Clin. Oncol..

[26] Shaw A.T., Kim D.W., Nakagawa K., Seto T., Crinó L., Ahn M.J., De Pas T., Besse B., Solomon B.J., Blackhall F. (2013). Crizotinib versus chemotherapy in advanced ALK-positive lung cancer. N. Engl. J. Med..

[27] Mahmood U., Huynh M.A., Killoran J.H., Qian J.M., Bent E.H., Aizer A.A., Mak R.H., Mamon H.J., Balboni T.A., Gunasti L. (2022). Retrospective Review of Outcomes After Radiation Therapy for Oligoprogressive Disease on Immune Checkpoint Blockade. Int. J. Radiat. Oncol. Biol. Phys..

[28] Ebadi M., Ladbury C., Liu J. (2023). Stereotactic Body Radiation Therapy for Oligoprogressive and Oligorecurrent Non-Small-Cell Lung Cancer. Clin. Lung. Cancer.

[29] Pacifico P., Colciago R.R., De Felice F. (2022). A critical review on oligometastatic disease: A radiation oncologist’s perspective. Med. Oncol..

[30] Kroeze S.G.C., Schaule J., Fritz C. (2021). Metastasis directed stereotactic radiotherapy in NSCLC patients progressing under targeted- or immunotherapy: Efficacy and safety reporting from the ‘TOaSTT’ database. Radiat. Oncol..

[31] Mok F.S.T., Tong M., Loong H.H. (2022). Local ablative radiotherapy on oligo-progression while continued on epidermal growth factor receptor tyrosine kinase inhibitors in advanced non-small cell lung cancer patients: A longer cohort. Asia Pac. J. Clin. Oncol..

[32] Yamamoto T., Niibe Y., Aoki M. (2020). Analyses of the local control of pulmonary Oligometastases after stereotactic body radiotherapy and the impact of local control on survival. BMC Cancer.

[33] Nicosia L., Franceschini D., Perrone-Congedi F. (2022). A multicenter LArge retrospectIve daTabase on the personalization of stereotactic ABlative radiotherapy use in lung metastases from colon-rectal cancer: The LaIT-SABR study. Radiother. Oncol..

[34] Franzese C., Comito T., Toska E. (2019). Predictive factors for survival of oligometastatic colorectal cancer treated with Stereotactic body radiation therapy. Radiother. Oncol..

[35] Rosner S., Liu C., Forde P.M., Hu C. (2022). Association of Pathologic Complete Response and Long-Term Survival Outcomes Among Patients Treated with Neoadjuvant Chemotherapy or Chemoradiotherapy for NSCLC: A Meta-Analysis. JTO Clin. Res. Rep..

